# ASO Author Reflections: Role of Local Treatment for Oligometastatic Esophagogastric Cancer

**DOI:** 10.1245/s10434-022-11546-9

**Published:** 2022-03-19

**Authors:** Tiuri E. Kroese, Peter S. N. van Rossum, Richard van Hillegersberg

**Affiliations:** 1 Department of Surgery, University Medical Center Utrecht, Utrecht University, Heidelberglaan 100, 3584 CX, Utrecht, The Netherlands; 2 Department of Radiation Oncology, University Medical Center Utrecht, Utrecht University, Heidelberglaan 100, 3584 CX, Utrecht, The Netherlands

## Past

The concept of oligometastatic disease (OMD) implies that radical local treatment for OMD (e.g., metastasectomy or stereotactic body radiotherapy [SBRT]) can improve overall survival (OS).^1,2^ This benefit of local treatment for OMD might be explained by the “seed and soil” hypothesis.^3^ This hypothesis suggests that metastatic spread is the result of the interaction between tumor cells and the target organ.^3^ According to this concept, certain tumors have a predisposition for a particular organ only because of this selective interaction.^3^ This process might explain why patients experience a limited number of metastases in a certain organ only and why radical local treatment to that organ improves OS.

Recent randomized controlled trials (RCTs) have indeed shown that local treatment of OMD improves survival outcomes for patients with prostate, colorectal, breast, and non-small cell lung cancer.^4–6^ However, patients with esophagogastric cancer were not included in these RCTs. Therefore, the optimal management for patients with oligometastatic esophagogastric cancer is unclear.

## Present

This study showed that for patients with oligometastatic esophagogastric cancer, local treatment of OMD plus systemic therapy was associated with a favorable prognosis (median OS, 35 months) and independently associated with better OS than either local treatment for OMD (median OS, 17 months) or systemic therapy alone (median OS, 16 months).^7^ This was mainly because of improved progression-free survival in the combined treatment group, probably due to a synergistic effect of the local and systemic control.

The results of this study are comparable with those of two prospective trials.^8,9^ The FLOT-3 trial by Al-Batran et al.^8^ included patients who had gastric or gastroesophageal junction adenocarcinoma with synchronous retroperitoneal lymph node metastases with or without metastases to one organ. After four cycles of fluorouracil, leucovorin, oxaliplatin, and docetaxel (FLOT) chemotherapy, the patients without progression underwent surgical resection of the primary tumor and metastases.^8^ This study showed an OS of 31.3 months for the patients who underwent systemic therapy and resection of the primary tumor and metastases compared with 15.9 months after systemic therapy alone.^8^ Another trial by Liu et al.^9^ included patients who had esophageal squamous cell carcinoma with three or fewer metachronous extra-regional lymph nodes or organ metastases. All the patients underwent SBRT, and 50% underwent four cycles of chemotherapy after SBRT.^9^ This study showed an OS of 24.6 months.^9^ Furthermore, the REGATTA trial has shown that for patients who have gastric cancer with synchronous OMD, systemic therapy plus resection of the primary tumor only (i.e., without resection of synchronous metastases) does not improve OS compared with systemic therapy alone.^10^ Altogether, the aforementioned studies suggest that the optimal management of synchronous OMD comprises resection of the primary tumor and metastases.

## Future

The authors believe future research should focus on two important aspects. First, the potential benefit of local treatment for OMD plus systemic therapy over either systemic therapy or local treatment alone requires confirmation. In that regard, results from the ongoing RENAISSANCE phase 3 trial by Al-Batran et al.^11^ are eagerly awaited. This trial addresses the potential benefit from surgical resection of the primary tumor and metastases plus systemic therapy over systemic therapy alone in gastric or gastroesophageal junction cancer patients who have synchronous retroperitoneal lymph node metastases with or without one organ with metastases. After four cycles of FLOT chemotherapy, patients without progression will be randomized to either additional chemotherapy or additional chemotherapy plus surgical resection of the primary tumor and metastases.^11^ In addition, an ongoing phase 3 trial by the National Cancer Institute addresses the potential benefits of radiotherapy plus systemic therapy over systemic therapy alone for gastric or esophageal cancer patients who have three or fewer radiologically visible metachronous metastases.^12^ After four cycles of CapOx or FLOT chemotherapy, patients without progression will be randomized to either additional systemic therapy or additional systemic therapy plus radiotherapy to the metastases.^12^

Importantly, the ongoing RCTs^11,12^ use various definitions and treatment strategies for oligometastatic esophagogastric cancer. A universal consensus definition of OMD in these patients could aid in the standardization of inclusion criteria in future clinical trials and prospective data collection. The OligoMetastatic Esophagogastric Cancer (OMEC) project aims to develop a multidisciplinary European consensus statement for the definition and strategy for treating oligometastatic esophagogastric cancer.^13^ This consensus statement can aid clinicians in decision-making and is expected to result in a prospective European trial for these patients.
